# Editorial: New Strategies in Design and Synthesis of Inorganic Pharmaceuticals

**DOI:** 10.3389/fchem.2020.00453

**Published:** 2020-05-28

**Authors:** Muhammad Hanif, Xiaoda Yang, Arthur D. Tinoco, Damian Plażuk

**Affiliations:** ^1^School of Chemical Sciences, The University of Auckland, Auckland, New Zealand; ^2^State Key Laboratories of Natural and Biomimetic Drugs, Department of Chemical Biology, School of Pharmaceutical Sciences, Peking University Health Science Center, Beijing, China; ^3^Department of Chemistry, University of Puerto Rico, San Juan, Puerto Rico; ^4^Department of Organic Chemistry, Faculty of Chemistry, University of Łódz, Łódz, Poland

**Keywords:** metallodrugs, anticancer agents, metalloenzymes, inhibitors, Alzheimer's disease

Metalions not only perform crucial functions in biology, but they are widely used for diagnostic and therapeutic agents applications. Examples of clinical uses include gadolinium complexes as MRI contrast agents, technetium-99m complexes as imaging agents and platinum-based anticancer agents drugs. DNA targeted platinum drugs are used in more than 50% of cancer treatments either alone or in combination therapy. Despite their huge success in the clinic, they are not devoid of drawbacks including severe side effects due to dose-related toxicity and the emergence of drug resistance. These limitations have inspired the investigation of platinum and non-platinum metal complexes with modes of action different from those of platinum drugs. As a result, the compounds of metals present across the periodic table were designed and developed for a range of diseases from cancer (e.g., Ru, Gd, Ti, Ge, V, and, Ga) to diabetes (V and Cr) to infectious diseases (Ag, Cu, and Ru). Each metal has unique features such as redox potentials and ligand exchange kinetics. Therefore, the choice of the metal center and ligand design play a crucial role in the therapeutic effects and the mechanism of action for new agents (Hanif and Hartinger, 2018).

This special issue “New Strategies in Design and Synthesis of Inorganic Pharmaceuticals” compiles six articles on the latest advances in areas of metallodrug discovery and development.

Half-sandwich metal-arene scaffold offers features that can be manipulated to optimize the drug-like properties of molecules. This class of compounds has attracted considerable interest in recent years. Mokesch et al. reported a series of novel half-sandwich Ru^II^ and Os^II^ complexes of 2-phenylbenzothiazole derivatives. The Ru^II^ and Os^II^ complexes exhibited anticancer activity in the low μM range. The 2-phenylbenzothiazole derivatives used as ligands were at least an order of magnitude less potent than the metallacycles. The article reports the aqueous stability, interaction with small biomolecules, cellular accumulation, and apoptosis/necrosis induction of the metallacycles. The fluorescence microscopy of representative Ru^II^ complex showed high accumulation in lysosomes and other subcellular compartments.

Molecularly targeted drugs have shown enormous potential in improving the unwanted side effects and toxicity of anticancer agents. The targeted drugs recognize and bind to the receptors overexpressed on the surface of cancer cells compared to the healthy cells. In this regard, epidermal growth factor receptor (EGFR), overexpressed in various tumors, has been explored extensively. Li et al. functionalized Pt^II^ terpyridine complexes with EGFR inhibiting 4-anilinoquinazoline derivatives. The anticancer Pt^II^ compounds demonstrated multiple modes of DNA interaction and were highly potent EGFR inhibitors. The results are very encouraging for the future design of multi-targeting agents.

The zinc-containing metalloenzymes including histone deacetylases, carbonic anhydrases, and matrix metalloproteinases are overexpressed in many tumors. Therefore, these cancer-associated metalloenzymes are considered an essential target in anticancer drug discovery programs. Ye et al. reviewed recent progress in the design of metal complexes as inhibitors of zinc-containing metalloenzymes. The obvious benefit of using metal complexes as enzyme inhibitors is their ability to construct three-dimensional shapes for fine-tuning the optimized enzyme-binding affinity and selectivity.

Liu et al. reported the synthesis of half-sandwich Ir^III^ N-heterocyclic carbene (NHC) complexes using pentamethylcyclopentadienyl derivatives as arene co-ligands. The complexes showed interesting antitumor properties against A549 and influenced the mitochondrial membrane potential. The complexes were evaluated with regard to their binding to bovine serum albumin, catalysis of the oxidation of nicotinamide adenine dinucleotide and induction of reactive oxygen species. The laser confocal experiment showed cellular uptake of Ir^III^ complexes, and their accumulation in the lysosome, ultimately leading the induction of apoptosis.

Vanadium compounds such as [V^IV^O(acac)_2_] are well-known for their antidiabetic and anticancer properties. Under physiological conditions, vanadium complexes undergo interconversion between +III and +V oxidation states, which facilitate binding to proteins. In this context, Sciortino et al. used lysozyme (Lyz) and ubiquitin (Ub) as model proteins to study the transformation of V^III^(acac)_3_, V^IV^O(acac)_2_, and V^V^O_2_${\left(\text{acac}\right)}_{2}^{-}$ by experimental and computational methods. The V^III^(acac)_3_ underwent oxidation and consequently formed n[V^IV^O(acac)]–protein and n[V^IV^O(acac)_2_]–protein adducts. The complexes V^IV^O(acac)_2_ and V^V^O_2_${\left(\text{acac}\right)}_{2}^{-}$ demonstrated dissociation to give mono-chelated species V^IV^O(acac)^+^ and V^V^${\text{O}}_{2}^{+}$ ion, respectively. These dissociation products then formed adducts with Lyz and Ub. Overall, the authors concluded that the V^IV^O(acac) complex was best at tolerating the presence of the investigated proteins. Its adducts with proteins or biomolecules could be responsible for its pharmacological properties, an important consideration in future design strategies.

Alzheimer's disease (AD) is a significant health concern, in particular, in the context of the aging population. One of the characteristics of AD is the formation of extracellular aggregates of the amyloid-beta (Aβ) peptide. Gomes et al. reported NAMI-A type Ru^III^ complexes in which the imidazole ligand was replaced by pyridine derivatives. The complexes were investigated as potential therapeutics for AD. The complexes bind covalently to the Aβ peptide. While the peptide alone leads to precipitation, however, the binding of Ru^III^ complexes causes the formation of soluble high molecular weight aggregates. The Aβ aggregation was not dependent on the size of the pyridine ligand. The results showed that the Ru^III^ complexes were capable of modulating Aβ peptide aggregation.

It has been a pleasure to edit this exciting topic of Frontiers in Chemistry. The issue brings together a wide variety of articles about medicinal applications of metal complexes. The editors hope that the articles will be of interest for researchers in the field of medicinal bioinorganic chemistry. The articles will contribute to improving the knowledge and understanding of the biological properties of metal complexes for future drug design.

## Author Contributions

All authors listed have made a substantial, direct and intellectual contribution to the work, and approved it for publication.

## Conflict of Interest

The authors declare that the research was conducted in the absence of any commercial or financial relationships that could be construed as a potential conflict of interest.
